# Modulation of Pectin on Mucosal Innate Immune Function in Pigs Mediated by Gut Microbiota

**DOI:** 10.3390/microorganisms8040535

**Published:** 2020-04-08

**Authors:** Weida Wu, Li Zhang, Bing Xia, Shanlong Tang, Jingjing Xie, Hongfu Zhang

**Affiliations:** 1 State Key Laboratory of Animal Nutrition, Institute of Animal Sciences, Chinese Academy of Agricultural Sciences, Beijing 100193, China; harrypolowwd87@163.com (W.W.); xiabingcaas@126.com (B.X.); long18763897938@163.com (S.T.); xiejingjing@caas.cn (J.X.); 2 State Key Laboratory of Food Science and Technology, School of Food Science and Technology, Nanchang University, Nanchang 330047, China; douweibahua@163.com

**Keywords:** pectin, pigs, mucosal immune, SCFAs, microbiota

## Abstract

The use of prebiotics to regulate gut microbiota is a promising strategy to improve gut health. Pectin (PEC) is a prebiotic carbohydrate that enhances the health of the gut by promoting the growth of beneficial microbes. These microbes produce metabolites that are known to improve mucosal immune responses. This study was conducted to better understand effects of PEC on the microbiome and mucosal immunity in pigs. Pigs were fed two diets, with or without 5% apple PEC, for 72 days. Effects of PEC on the microbiota, cytokine expression, short-chain fatty acids (SCFAs) concentration and barrier function were examined in the ileum and cecum of the pigs. An integrative analysis was used to determine interactions of PEC consumption with bacterial metabolites and microbiome composition and host mucosal responses. Consumption of PEC reduced expression of pro-inflammatory cytokines such as IFN-γ, IL-6, IL-8, IL-12 and IL-18, and the activation of the pro-inflammatory NF-κB signaling cascade. Expression of *MUC2* and *TFF* and the sIgA content was upregulated in the mucosa of PEC-fed pigs. Network analysis revealed that PEC induced significant interactions between microbiome composition in the ileum and cecum on mucosal immune pathways. PEC-induced changes in bacterial genera and fermentation metabolites, such as *Akkermansia*, *Faecalibacterium*, *Oscillibacter*, *Lawsonia* and butyrate, correlated with the differentially expressed genes and cytokines in the mucosa. In summary, the results demonstrate the anti-inflammatory properties of PEC on mucosal immune status in the ileum and cecum effected through modulation of the host microbiome.

## 1. Introduction

Consumption of dietary fiber is associated with amelioration of intestinal inflammation, which occurs via modulation of the microbial community composition and enhancement of the production of short-chain fatty acids (SCFAs) [1,2]. Fiber modulates the crosstalk between the gut microbes and the intestinal mucosa. This cross-communication is mediated by interactions between the mucosa and microbial metabolites and microbiota-associated molecular patterns (MAMP) [3,4,5]. SCFAs, and in particular, butyrate, are an essential fuel for intestinal epithelial cells and are known to have crucial immunomodulatory properties [6]. SCFAs are known to signal through cell-surface G-protein-coupled receptors (GPCRs), like GPR41 and GPR43, to activate signaling cascades that regulate intestinal immune functions [7]. The microbiome is a source of multiple immunogenic molecules, such as lipopolysaccharide (LPS) and lipoteichoic acids (LTA), which are able to activate the immune system through activation of pattern-recognition receptors, such as Toll-Like Receptors (TLRs) [4,8].

Pectin (PEC) is a molecule with an α (1-4)-linked galacturonic acid backbone and can differ in degrees of methyl esterification (DM). It has been reported to ameliorate intestinal inflammation by downregulating the expression of inflammatory mediators, such as pro-inflammatory IL-6, IL-8 and NF-κB [9,10], and enhancing the expression of *MUC2*, which decreased bacterial adherence to the epithelial cell surface and reduced susceptibility to colitis [11]. Broken mucus layers resulting from a lack of Muc-2 mucin result in increased intestinal permeability, and they are more likely to develop spontaneous colitis [12,13]. Increased SCFAs production with pectin consumption may provide the most direct link between pectin intervention and associated gut health benefits [14]. PEC is found to stimulate growth and activity of bacterial genera, such as *Prevotella*, *Lactobacillus* and *Faecalibacterium* [15,16,17], members of which have been shown to promote gut health [15,18]. In pigs, we recently showed that PEC intake significantly affected ileal and cecal secondary bile acids metabolism, which was closely related to changes in the intestinal microbiota and may have consequences for microbe–host signaling [19]. Many studies have investigated PEC-related alteration in luminal or fecal microbiota; however, we previously showed that alteration of mucosal associated microbiome may be a very important mechanism of PEC effect on immune function [20]. However, little is known about PEC-related alteration of mucosal microbiome, mucosal immune status and its effects on the barrier function. Furthermore, as the primary site of PEC fermentation is the colon, little is known about its effects in the small intestine.

We hypothesized that the intake of PEC would modulate the gut microbiota and their metabolic products and that these changes would benefit host intestinal issues. Therefore, we investigated the effect of PEC consumption on the cytokine and expression of inflammatory genes, barrier function and mucosal defense in the ileum and cecum of pigs. Integrative analysis of the cytokine, expression of genes and specific bacteria or SCFAs were performed to determine the mechanisms of the dietary alteration-microbial interactions that may contribute to the enhancement of mucosal immunity by PEC.

## 2. Materials and Methods

### 2.1. Experimental Design and Animals

The detailed experimental design and animals are described in Fang et al. [19], and intestinal samples used in the present study were obtained from the same pigs. Briefly, twelve Duroc × Large White crossbred piglets (initial body weight: 11.05 (SD 0.11) kg) were randomly assigned to one of two diets, in a randomized design. Each group had six pigs. Pigs were individually fed for 72 days and had ad libitum access to deionized water (nipple drinkers). Before the experiment, pigs were fed with a commercial diet in a 7-d environmental adaptation.

Each group of piglets was fed with corn–soybean meal diets containing 5% apple pectin (PEC, purchased from Yuzhong Biotech Corporation, Henan, China) or cornstarch (purchased from Yufeng Cornstarch and Sugar Company, Hebei, China) as the control (CON). The diets (Table S1) had been formulated to meet the nutritional requirements suggested by NRC (2012) for pigs within the corresponding weight range (Table S1) [21]. The piglets were manually fed twice each day, at 8:30 and 15:30. Feed allowances were calculated to meet the 1.13-fold digestible energy requirement. The amount of feed supplied was adjusted weekly. All experimental procedures were approved by the Animal Welfare Committee in the Institutes of Animal Sciences, Chinese Academy of Agricultural Sciences, on 4 May 2017 (Ethics Code Permit IAS2017-3), and were compliant with the Regulations for the Administration of Affairs Concerning Experimental Animals (The State Science and Technology Commission of P. R., China, 1988).

### 2.2. Sample Collection

Blood samples were collected from the anterior vena cava before the morning meal, on the last day of the experiment. Pigs were sampled two hours after the morning feeding on day 72. Pigs were humanely euthanized by electrical stunning and exsanguination. Thereafter, the abdominal cavity was opened for tissue collection. The ileum and cecum were opened for collection of luminal contents, which were then homogenized and stored at −80 °C, for later SCFAs quantification. These intestinal segments were washed in ice-cold PBS, and the mucosa was scraped off, using a glass microscope slide. Mucosa samples were immediately snap-frozen and stored at −80 °C, for subsequent bacterial community analysis and mRNA measurements.

### 2.3. Chemical Analysis

The ileal and cecal mucosa (0.1 g) were mixed with 1 mL of PBS and homogenized. The sIgA concentration in the homogenate was determined by using a swine ELISA kit (Cusabio Biotech Co., Ltd., Hubei, China), according to the manufacturer’s instructions. Serum diamine oxidase (DAO), D-lactic acid and alkaline phosphatase (AKP) concentration were analyzed, using commercial kits (Jiancheng Bioengineering Institute, Nanjing, China), following the protocols described by the manufacturers. The composition of SCFAs was analyzed by using gas chromatography (GC), based on our previous methods [22].

### 2.4. Measurement of Cytokine Concentrations in Mucosa

Cytokine concentrations in the mucosa were quantified by using the Luminex xMAP R technology, a multiplexed microsphere-based flow cytometric assay, according to the manufacturer’s instructions. Precoated magnetic beads for pig reactivity (Merck Millipore, Burlington, MA, USA) were used for the determination of GM-CSF, interferon-gamma (IFN-γ), interleukin (IL)-1a, IL-1b, IL-1Ra, IL-2, IL-4, IL-6, IL-8, IL-10, IL-12, IL-18 and TNF-α. 

### 2.5. Quantitative Real-Time PCR Analysis

Total RNA was extracted from the ileal and cecal mucosa, using the RNeasy Mini Kit (Qiagen, Hilden, Germany). The integrity of RNA was checked by electrophoresis, before reverse transcription. The cDNA was transcribed by using the High-Capacity cDNA Archive kit (Takara, Takara Biomedical Technology in Beijing, China). RNase free condition was maintained throughout the whole process. Real-time PCR was carried out on an ABI 7500 real-time PCR system (Applied Biosystems, Waltham, MA, USA), and an SYBR Premix Ex Taq II (Takara, Takara Biomedical Technology in Beijing, China) was used. The mRNA levels of GAPDH and β-actin were used as internal control. All primer sets are provided in Supplementary Table S2. Relative gene expression was calculated relative to the pig with the lowest expression of the respective gene, using the 2^−ΔΔ^Cq method [23,24].

### 2.6. DNA Extraction, PCR Amplification and Illumina HiSeq Sequencing

Total bacterial DNA was extracted from the mucosa of pigs, using the Qiagen DNA isolation kit (Qiagen, Hilden, Germany), according to the manufacturer’s instructions. For 16S ribosomal RNA (rRNA) gene-based microbial composition profiling, barcoded amplicons from the V3–V5 region of 16S rRNA genes were generated by a 2-step PCR protocol, and the 16S rRNA gene was sequenced on the Illumina HiSeq sequencing platform, as described before [25]. Raw Illumina fastq files were demultiplexed, quality filtered and analyzed, using QIIME 1.8.0 [26], as described previously [25], while using the Silva 123 reference database. Predictive functional profiling of microbial communities was conducted by Phylogenetic Investigation of Communities by Reconstruction of Unobserved States (PIRCUSt) [27].

### 2.7. Statistical Analyses

Data on serum DAO, D-lactic acid and AKP, intestinal tissue sIgA, cytokines, bacterial α-diversity indices (Chao1, and Shannon), SCFAs and gene expression were analyzed by the Tukey–Kramer test and the Duncan multiple comparison method (JMP 13.0, SAS Institute, Inc., Cary, NC, USA). Results are expressed as least-square means with standard error of the mean. Statistical significance was set at *p* < 0·05, whereas a trend was declared at 0·05 < *p* ≤ 0·10.

All statistical analyses of operational taxonomic unit (OTU) reads were conducted in the R program (V3.6.1, available online: https://www.r-project.org/). Non-metric multidimensional scaling (NMDS) based on Weighted-Unifrac distance matrices was performed to obtain β-diversity between the groups. A permutational multivariate ANOVA test was calculated on the Weighted-Unifrac matrices, using 999 random permutations and at a significance level of 0.05. Pairwise comparisons based on a negative binomial Wald test from the DESeq2 software package was used to measure the dietary differences in individual OTUs at the different taxon level [28,29]. A BH-corrected *p*-value of 0.05 was considered statistically significant [30]. 

The relevance network analyses in the ‘mixOmics’ package were used to integrate the mucosal gene expression, bacterial genera and SCFAs in the ileum and cecum [31,32]. Partial Spearman’s correlation coefficients were computed, to identify the discriminate bacteria with the identified genus and SCFAs in relation to the mucosal gene expression in the ileum and cecum.

## 3. Results

### 3.1. Growth Performance, Nutrient Digestibility and Serum Parameters 

**Figure 1:**
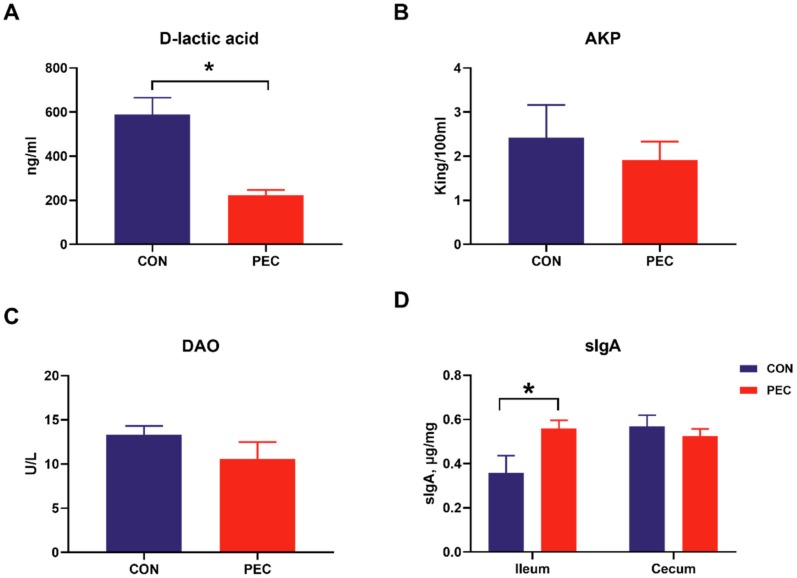
The concentration of D-lactic acid (**A**), AKP (**B**) and DAO (**C**) in serum and sIgA content (**D**) in intestinal mucosa of pigs fed either the control or pectin diet. Values are means (n 6/diet), with their standard errors represented by vertical bars. * Means statistically significant (*p* ≤ 0·05).

No intestinal and systemic disorders were observed during the experimental period; all pigs were clinically healthy. The average daily weight gain was similar and amounted to 599.83 and 651.83 (31.74) g for the CON and PEC diets, respectively. Similarly, pigs had a similar average feed-to-gain ratio (F: G) (2.23 and 2.18 (0.077)) for CON- and PEC-fed pigs, respectively. D-lactic acid is the product of inherent bacteria in gastrointestinal tract, and the levels of DAO and AKP in the blood are also considered as proper indicators of intestinal permeability and inflammatory response [33,34,35]. The pigs fed with pectin had lower plasma D-lactic acid concentration (*p* < 0.05) than pigs fed with the control diet, whereas the AKP and DAO were similar between PEC- and CON-fed pigs (Figure 1A–C).

### 3.2. Pectin Intervention Alters Bacterial Microbiome in Ileal and Cecal Mucosa

**Figure 2:**
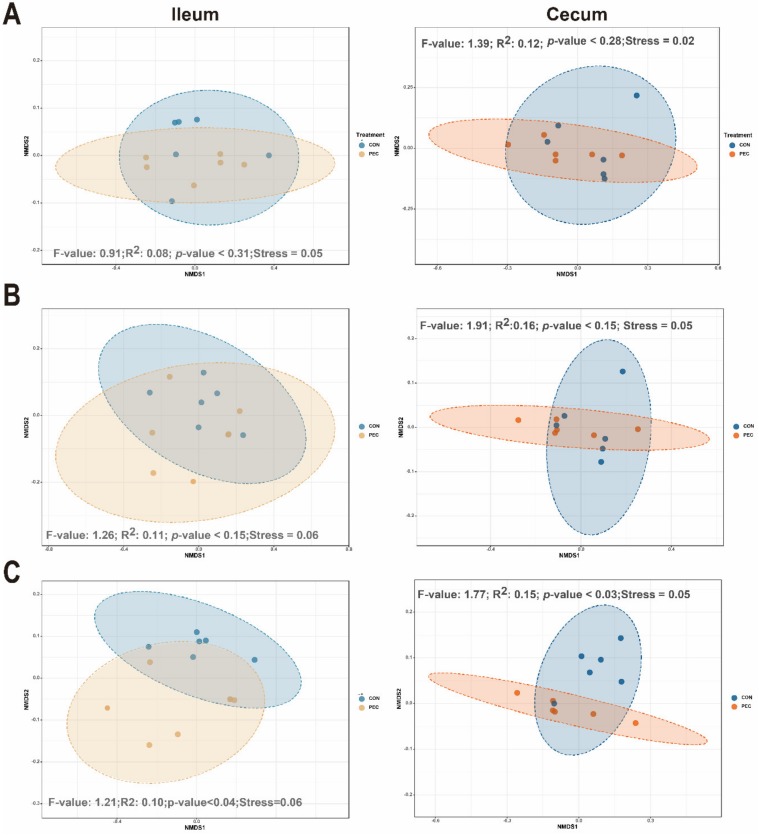
Effect of pectin on bacterial community in mucosa of growing pigs. Distances created with weighted-Unifrac distance show ileal or cecal mucosa at different taxonomy levels: Phylum (**A**), Family (**B**) and Genus (**C**). The *p*-value represents diet differences among NMDS scores along component 1. CON, control; PEC, pectin.

Samples were rarefied to 25,648 reads, to account for unequal numbers of sequences between samples, before calculating the α-diversity index. In ileal mucosa, PEC-fed pigs showed significantly lower diversity at the genera levels for Chao1, relative to pigs assigned to the control diet (Table S3). No dietary differences were seen in cecal mucosa by using Chao1 diversity measurements. For the Shannon index, no differences were noted at any of the taxonomic levels between the diets in the ileum and cecum (Table S4). At the genus level, dietary discrimination of samples was not as apparent in nonparametric multidimensional scaling (NMDS) from ileal samples. However, based on analysis of group similarities (ANOSIM) assessment on weighted-Unifrac-derived dissimilarity matrices at genus level, pectin significantly altered the community structure of the microbiome in the ileum. Similar ordination results were found for cecum samples (Figure 2).

The microbial composition, at the phylum and genus levels, was investigated in ileal and cecal mucosa. The phylum *Firmicutes* was the most abundant in the ileal and cecal mucosa of pigs. Abundance of 4 of the 24 phyla detected were significantly altered by PEC in the ileum. The abundance of *Tenericutes* was higher in PEC-fed pigs. On the other hand, *Bacteroidetes*, *Proteobacteria* and *Chlamydiae* were less abundant in PEC-fed pigs compared to CON. Similar shifts within the *Chlamydiae* were seen in the cecal mucosa. A total of 82 genera differed significantly between treatments, and these ranged in relative abundance, from 0.004% to 13.5%. Only four genera were consistently affected by PEC in the two gut sites, including *Lawsonia, Bacteroides, Staphylococcus* and *Aeromonas*, and their relative abundance was decreased with PEC consumption. All the genera that were significantly different (*p* < 0.05) are reported in Table S5.

Most of the treatment differences at the genus level occurred in the ileal mucosa. Fifty-four genera differed significantly between treatments but were mainly present at low relative abundances. Most genera affected by the PEC diet belonged to the phylum *Proteobacteria*, including *Helicobacter, Lawsonia, Stenotrophomonas*, *Pseudoalteromonas, Parasutterella, Rhodobacter,* etc. The relative abundances of *Akkermansia, Mycoplasma*, *Enterococcus* and *Propionibacterium* were lower in ileal mucosa due to pectin supplementation, whereas *Parabacteroides, Lactococcus* and *Ruminiclostridium_5* were more abundant. In the cecal mucosa, *Anaeroplasma*, *Anaerostipes*, *Faecalibacterium*, *Prevotella, Lachnoclostridium, Oscillibacter, Lachnospiraceae_UCG_004, Ruminiclostridium and Clostridium_sensu_stricto_6* were more abundant in PEC-fed pigs, relative to the CON-fed group, while *Chlamydia*, *Escherichia_Shigella*, *Streptococcus*, *Actinobacillus* and *Klebsiella* were lower in the PEC-fed group.

### 3.3. PEC-Associated Effects on Ileal and Cecal Microbial Metabolites

**Figure 3:**
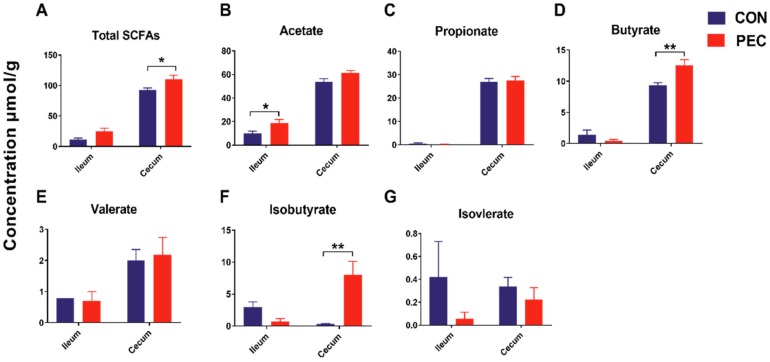
Short-chain fatty acids (SCFAs) concentrations (μmol/g) in ileum and cecum of growing pigs fed a diet with or without a 5% pectin supplement. (**A**) Total SCFAs, (**B**) Acetate, (**C**) Propionate, (**D**) Butyrate, (**E**) Valerate, (**F**) Isobutyrate, (**G**) Isovalerate. Total SCFAs are the sum of the following SCFAs: acetate, propionate, isobutyrate, butyrate, isovalerate and valerate. Group differences were tested with a Tukey–Kramer test. ** *p* < 0.01; * *p* < 0.05. CON, control; PEC, pectin.

Total SCFA concentrations were 1.18-fold higher (*p* < 0.05) in the cecum with PEC consumption, compared to the CON diet. Individual SCFA profiles showed 1.34-fold more butyrate and 22.5-fold greater iso-butyrate concentrations in the cecum, whereas they showed 1.89-fold more acetate in the ileum (Figure 3).

### 3.4. PEC-related Alteration in Predicted Bacterial Metagenomic Functions

To better comprehend the functional roles of the microbiome and its metabolites, we used PICRUSt to investigate the functional profiles of intestinal bacterial community. The results demonstrated that the relative abundances of the genes involved in immune-related KEGG pathway were significantly changed by PEC consumption. A total of nine predicted metagenomic function genes of the mucosal microbiota in the ileum were differentially regulated between the CON- and PEC-fed pigs. As such, PEC-fed pigs had a higher relative abundance of the KEGG pathway genes “Cell motility and secretion”, “RNA transport”, “Primary immunodeficiency” and “Phosphotransferase system (PTS)”, as compared to CON-fed pigs (*p* < 0.05). In contrast, the predicted pathways “Staphylococcus aureus infection”, “Vibrio cholerae infection”, “Adipocytokine signaling pathway”, “Bisphenol degradation” and “PPAR signaling pathway” were lower in PEC pigs, as compared to CON pigs (*p* < 0.05) (Figure S1A).

In the cecum, we found that the KEGG pathway genes “Pathogenic Escherichia coli infection”, “Caffeine metabolism”, “Bacterial invasion of epithelial cells”, “Bacterial toxins” and “Influenza A” were less abundant in PEC-fed compared to CON-fed animals (*p* < 0.05). On the contrary, “Lysosome”, “Mineral absorption” and “Steroid hormone biosynthesis” were markedly increased with PEC consumption compared to the CON diet (Figure S1B). The altered immune-related functional pathway provides strong evidence for pectin to regulate mucosal immunity through microbiota.

### 3.5. Intestinal Cytokines

**Figure 4:**
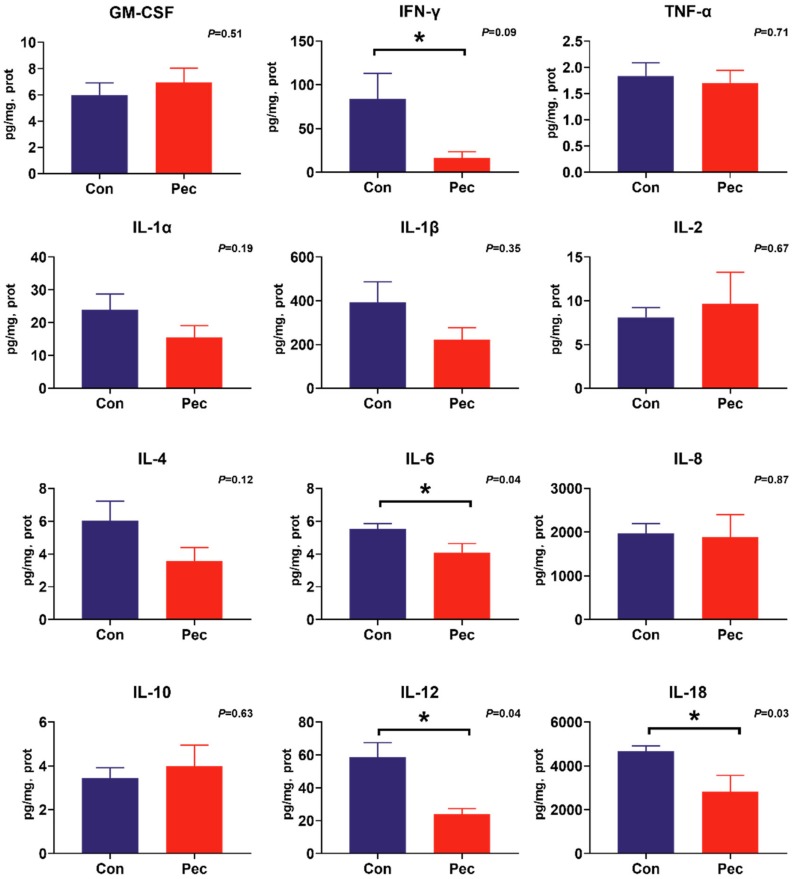
The concentration of cytokine in ileal mucosa of pigs fed either the control or pectin diet. Values are means (*n* = 6/diet), with their standard errors represented by vertical bars. * Means statistically significant (*p* ≤ 0·05).

**Figure 5:**
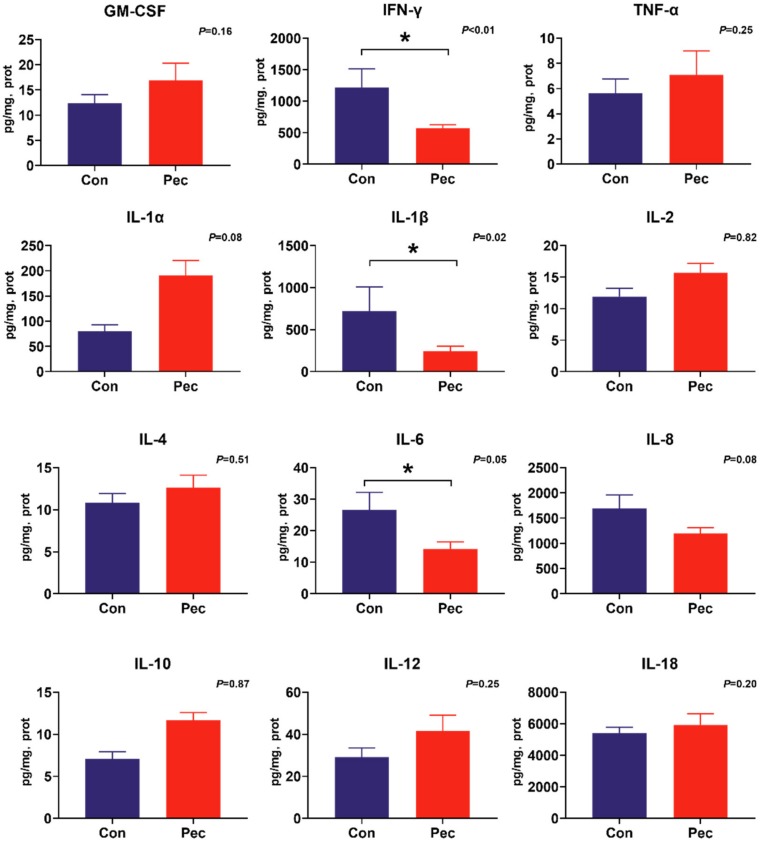
The concentration of cytokine in cecal mucosa of pigs fed either the control or pectin diet. Values are means (*n* = 6/diet), with their standard errors represented by vertical bars. * Means statistically significant (*p* ≤ 0·05).

To investigate the potential immunomodulatory properties of dietary PEC on immune status, the concentrations of the battery of cytokines were measured by cytokine magnetic beads. From this analysis, we found PEC intervention significantly reduce IL-12 (*p* = 0.04) and IL-18 (*p* = 0.03) concentrations, and tend to have reduced IFN-γ (*p* = 0.09) concentration in the ileal mucosa (Figure 4). In the cecal mucosa, significantly lower concentrations of IL-1β (*p* = 0.02) and IFN-γ (*p* = 0.02) were observed, as well as a trend toward increased concentration of IL-1α (*p* = 0.08) and IL-8 (*p* = 0.08). Intriguingly, consumption of the PEC diet decreased IL-6 concentration in both the ileal (*p* = 0.04) and cecal (*p* = 0.05) mucosa (Figure 5).

Intestinal slgA is another important host-produced protein that inhibits microbial pathogen adhesion; hence, ileal and cecal mucosa were assayed for total sIgA concentration. PEC-fed pigs had a higher concentration of ileal mucosa sIgA (*p* = 0.04), but there was no difference in sIgA concentration in the cecal mucosa (*p* > 0.20) (Figure 1D).

### 3.6. Mucosal Gene Expression

We conducted an analysis of the effects of pectin on intestinal gene expression linked to the innate immune response. This included the non-specific mucosal defense (mucin 2 (*MUC2*), mucin 4 (*MUC4*) and trefoil factor family (*TFF*)), barrier function (zonula occludens-1(*ZO-1*), *claudin-2*, *occludin* and myosin light chain kinase (*MLCK*)), apoptosis-related genes (B-cell lymphoma-2 (*BCL-2*), *bax* and *caspase3*), g-protein receptors (*GPR41* and *GPR43*), pathogen-recognition receptors (Toll-like receptors 2 and 4 (*TLR2* and *TLR4*)) and transcription factors (*NF-KB*, AMP-activated protein kinase (*AMPK*), TNF receptor associated factor 6 (*TRAF6*) and TGF beta-Activated Kinase 1 (*TAK1*)). 

In the ileum, supplementation of PEC tended to upregulate the expression of *MUC2* (*p* < 0.10) and significantly upregulated the expression of *claudin-2* (*p* = 0.04), whereas it downregulated the expression of *TLR2* (*p* < 0.10) and *NF-KB* (*p* < 0.10) as trends, compared with the CON diet (Table 1). 

The relative expression of *MUC2* (*p* = 0.04), *TFF3* (*p* < 0.01), *AMPK* (*p* < 0.01) and *TAK1*(*p* = 0.04) was upregulated in the cecal mucosa of pigs fed the PEC diet, compared with CON, while there was a tendency (*p* < 0.10) for higher *GPR41* expression in PEC fed pigs. Expression of *NF-KB* (*p* < 0.01) was downregulated by PEC, compared with the CON (Table 2).

### 3.7. PEC Modulates the Relevance Network between Relative Expression of Mucosal Genes, Bacterial Members and Metabolites

**Figure 6:**
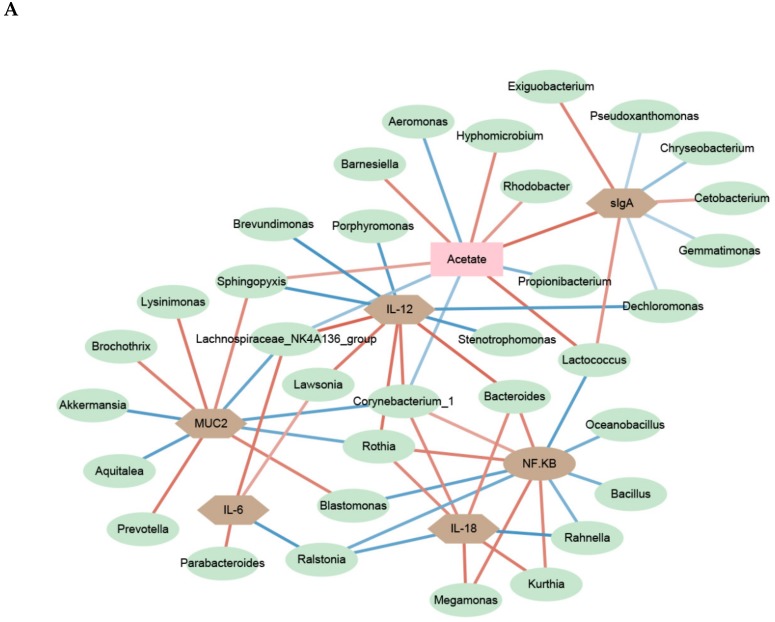

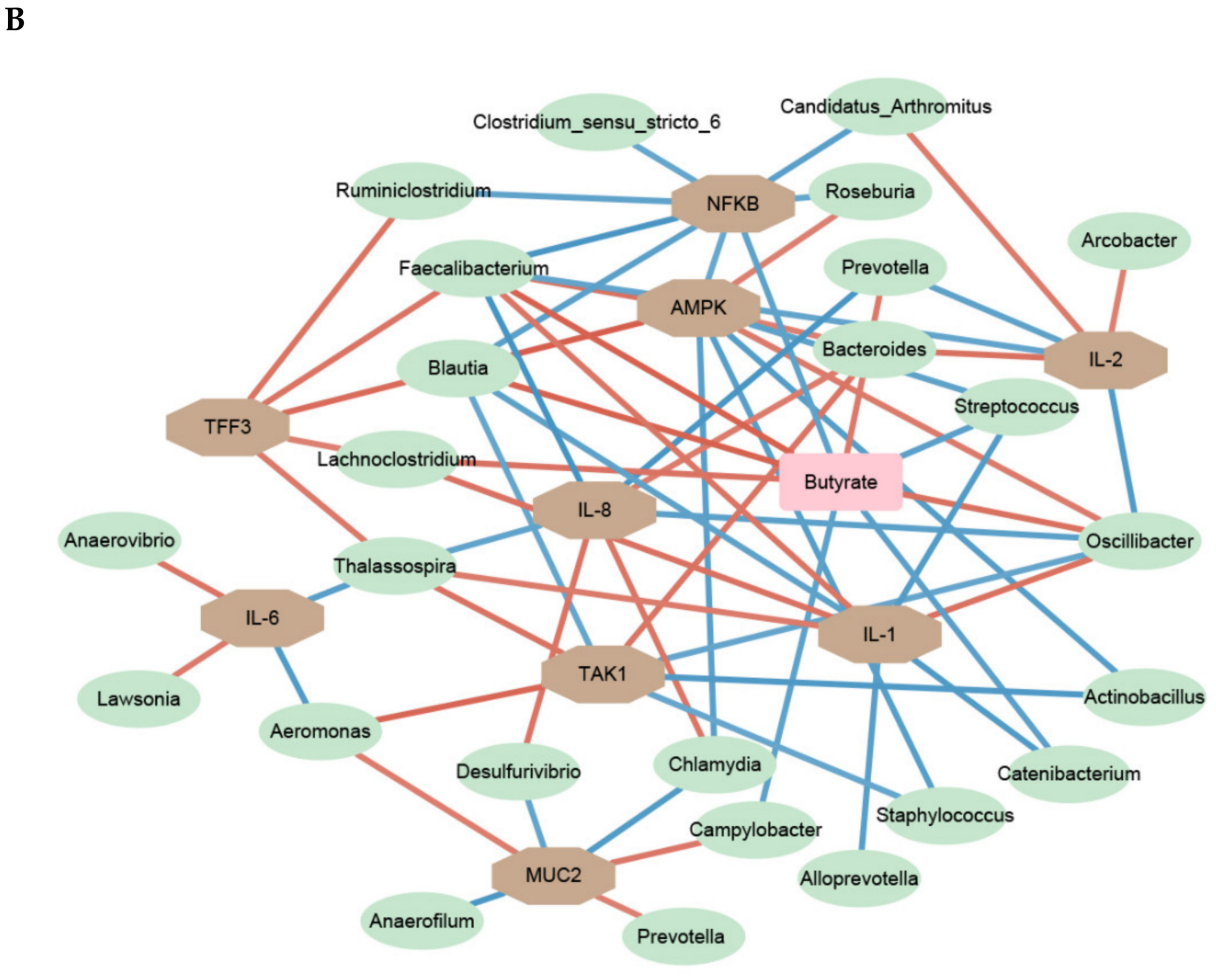
A network depicting correlations among cytokine contents, the relative abundances of bacterial genera, SCFA concentrations, gene expression, and sIgA concentrations in ileal (**A**) and cecal (**B**) mucosa of pigs using relevance networking. Only correlations with a partial spearman’s coefficient > 0.7 and a *p* < 0.05 are shown. Bacterial nodes are OTUs labeled with their taxonomic classification, according to the SILVA database. The edge color intensity indicates the level of the association: red, positive; blue, negative. Node shape indicates whether it is the mucosal immune (octagon), SCFAs(rectangular), or genera (ellipse).

As shown in Figure 6, to investigate relationships between bacterial genus, bacterial metabolites and host immune status in the mucosa, relevance network association analysis was constructed by using the relative abundance of genera, SCFA content, cytokine concentration, mucosal gene expression and sIgA concentrations. This relevance network model has proven effective in discovering significant interacting factors [22].

As shown for the ileum in Figure 6A, the differentially expressed genes and cytokines with the PEC diet positively or negatively associated with many bacterial genera and SCFA. For instance, *Bacteroides abundance* was positively associated with expression levels of *NFKB*, and IL-12 and IL-18 concentration. *Akkermansia* and *Aquitalea* levels were negatively correlated to the expression of *MUC2*, while *Prevotella, Brochothrix* and *Lysinimonas* were positively correlated. *Lactococcus* was positively correlated with the expression of *NFKB* and negatively correlated with acetate or sIgA concentrations.

The relevance network of cecal mucosa was more complex than that of ileal mucosa. The various bacterial genera affecting mucosal immunity were identified as butyric-acid-producing bacteria. Like the ileum, *Prevotella* was positively correlated with the expression of *MUC2*. The relative expression of *AMPK* in the ceca was positively associated with five genera: *Oscillibacter, Bacteroides, Faecalibacterium, Roseburia* and *Blautia.* Moreover, there were strong negative correlations between *Faecalibacterium, Oscillibacter, Roseburia and Blautia* and the expression of *NFKB. Lawsonia* was found to be closely associated with an increased concertation of IL-6 in both the ileal and cecum mucosa.

## 4. Discussion

Effects of PEC on microbiome composition in the gastrointestinal content in pigs has been shown in several studies [15,36,37]. However, there is a dearth of information on the impact of PEC on mucosal microbiota and immunity. The present results underscore the potential of PEC to modify the expression of genes and cytokine related to the innate immunity in the ileal and cecal mucosa of clinically healthy non-challenged pigs. Furthermore, these results demonstrate the presence of specific microbiome signatures on mucosal immune status in the ileal and cecal mucosa. This is evidenced by the finding that specific bacterial genera and fermentation metabolites were affected by pectin consumption, and the strong association of these changes with differently expressed host genes in the ileum and cecum. Although the primary site of action of PEC is the large intestine [13], comparable immunomodulatory effects on mucosal immunity were observed in both ileum and cecum of pigs. These results underline the importance of PEC-related microbial alteration in mucosal immune signaling modulated by PEC, in both the large and small intestine.

There are distinct differences in the microenvironment of the lumen and mucosal surfaces [20,38]. Mucosal-associated bacteria generally compose of oxygen-tolerant populations and mucolytic species adept at living on host glycoproteins, compared to digesta-associated microbes in the lumen. Proximity to the mucosal layer exposes the mucosa-associated microbiome to a host-derived oxygen source [38,39], stimulating the growth of oxygen-tolerant taxa, such as the *Proteobacteria*. The phylum *Proteobacteria* contains many potential opportunistic pathogens, including *Escherichia, Salmonella, Campylobacter* and *Helicobacter,* and its increase can be considered as a potential indicator of intestinal diseases [40,41]. In this study, we found that a PEC-related decrease of the *Proteobacteria* in the ileal mucosa might be a mechanism by which PEC promotes the gut health of pigs. An abundance of multiple specific bacterial groups was found to be significantly influenced by PEC in both ileum and cecum. PEC-enriched bacteria in the mucosa are mainly organisms known to use fermentative metabolisms. Well-known fermenters, such as *Lactococcus* and *Faecalibacterium*, were enriched in the PEC-fed pigs and are associated with intestinal health [42,43]. Other studies have shown an increased abundance of these genera in pigs fed pectin [16]. We also found the increased level of *Prevotella* with the PEC diet; *Prevotella* is associated with the consumption of a dietary fiber-rich diet [44] and has been suggested to be an anti-inflammatory microbe in animal gut [45]. Though they were clinically healthy, the CON group exhibited signs of reduced immune tolerance and higher abundances of potential bacterial pathogens in their mucosal tissues, relative to the PEC group. The bacteria enriched in the CON pigs, including *Lawsonia, Bacteroides, Staphylococcus* and *Streptococcus*, have previously been associated with inflammatory diarrhea and gut dysbiosis in pigs [46,47,48]. Notably, members of the genus *Bacteroides* have been shown to induces IL-8 secretion in intestinal epithelial cells via activation of the β-catenin pathway [49]. Our results showing correlations between the abundance of *Bacteroides* and IL-8, IL-12 and IL-18 suggest that these pro-inflammatory cytokines may also, in turn, play a role in expanding the niche for *Bacteroides* in the CON-fed animals. Additionally, we found the boom of *Helicobacter* in the ileal mucosa from CON pigs. Bacteria from this genus can be facultative intracellular pathogens, and it has been reported that *non-H. pylori Helicobacter* may be one of the causes of inflammatory bowel disease (IBD) [50]. These observations indicate that, despite the lack of clinical symptoms of illness in the two treatment groups, the mucosae of PEC fed pigs were less amenable to colonization and infection by pathogens relative to the CON pigs. Intriguingly, our data suggest that predicted functional alteration of the mucosal bacterial community by PEC consumption was characterized by the significantly decreased immune-associated metabolic pathways (including Staphylococcus aureus infection, Vibrio cholerae infection, Pathogenic Escherichia coli infection and Bacterial toxins), suggesting that a PEC diet helps in eliminating pathogen bacteria adhesion and thus may decrease the risk of immune response disorders.

The production of SCFA depends on the fermentation of PEC by the microbiota. In this study, it depended on a significant increase in butyrate in the cecum of PEC-fed pigs. This may be mainly due to the increase in abundance of some butyric acid-producing bacteria by PEC, including *Faecalibacterium*, *Lachnoclostridium, Blautia* and *Clostridium_sensu_stricto_6.* Butyrate is a recognized metabolite of bacteria that is essential for gut homeostasis that supports many aspects of gut health, including the downregulation of inflammatory cytokine expression in intestinal mucosal cells, enhancing barrier function and increasing immune system effector mechanisms [2,6]. PEC consumption also promoted the content of total cecal SCFA, which are rapidly oxidized by the intestinal epithelium. This reduces available oxygen concentrations, creating an environment that is more conducive to microbial fermentation [51]. Although SCFA-sensing G-protein-coupled receptors play an essential role in intestinal immune regulation [52], we did not find significant changes in G-protein-coupled receptors expression in the ileal and cecal mucosa. Increased beneficial bacteria population and metabolites in PEC-fed pigs will positively affect mucosal barrier immunity, as well as affecting mucosal cytokine concentration. Abundance of some proinflammatory cytokines, such as IL-1β, IL-8 and IL12, declined in the mucosa of pigs fed PEC. In addition, IL- 6 was reduced in the ileum and cecum in pigs fed PEC. A recent study reported that IL-6, as a pro-inflammatory cytokine, could induce intestinal epithelial barrier dysfunction and play a role in the pathological process of inflammatory bowel disease (IBD) [53]. Intriguingly, *Lawsonia* was previously found to be significantly associated with the increase of IL-6 [54,55]; thus, a potential link between Lawsonia and IL-6 expression is further suggested by our current findings, although the mechanism behind this association remains to be established. Besides, in a mice model of acute pancreatitis, low-methoxyl PEC was found to downregulate TNF-α, IL-1β and IL-6 mRNA levels in ileal and colonic tissue, leading to the recovery of acute pancreatitis-associated disorder of the intestinal barrier [56]. Furthermore, apple-derived pectin could reduce IL-6 expression in ileum tissue of mice fed with high-fat diets, suggestive of anti-inflammatory activity of PEC [10]. 

The effects of PEC supplementation on mucosal defense capacity was studied by assessing Muc2, TFF and sIgA. Mucin protein synthesized by goblet cells, coupled with crosslinking provided by TFF and Fc-γ binding proteins with MUC2 domains, results in a highly viscous extracellular layer [57]. However, results for ileal and cecal mucosa goblet cell numbers between PEC and CON pigs were inconclusive (data not shown). Besides, no correlations were found between SCFAs and *MUC2*. Recent studies demonstrated that low-methoxyl PEC stimulates small intestine mucin secretion irrespective of goblet cell proliferation and SCFAs modulation [11], and this is in line with our observations. It could be attributed to the PEC molecule, which could interact directly with epithelial tissue and promote mucin discharge from individual goblet cells accompanied by MUC2 upregulation [11]. Further studies are needed to clarify this direct “PEC-regulated” secretion. Besides, a number of bacterial taxa were positively associated with the downregulated cecal expression of MUC2 (e.g., *Akkermansia, Anaerofilum, Chlamydia* and *Helicobacter*). Two potential mechanisms might explain this association. First, some of these genera can degrade mucus and use it as a source of nutrients [58]. On the other hand, they can modify host glycan expression following invasion [59]. Feeding PEC upregulated the ileal expression of *TFF3* in the present study. A comparable downregulation in the expression of *TFF3* was observed in pigs challenged with *Trichuris suis*, receiving soluble fiber in the form of 10% inulin [60]. Absolute production of sIgA in the intestine exceeds the sum of all other antibody classes, and sIgA can eliminate pathogenic bacteria by stimulating mucosal layer formation. In a rodent model, higher fecal sIgA concentrations were found in PEC-supplemented mice, compared to cellulose-supplemented groups [61]. The significant correlation observed between the concentration of sIgA and acetate (*p* < 0.04) revealed that SCFAs stimulated sIgA production in response to PEC. Acetate has been reported to promote SIgA production by inducing DCs to express Aldh1a2, which converts vitamin A into retinoic acid (RA) [62]. The results of this study indicated that the increased expression of *MUC2* and *TFF*, and sIgA production, was accompanied by improved barrier integrity of PEC-fed pigs.

Modification in cytokine production and innate immune gene expression may be attributed to the altered mucosal microbiota and subsequent alteration in abundance of different microbe-associated MAMPS. The interaction of TLR with their MAMP induces NF-κB signaling, which influences expression of cytokines, defensins and MUC2 [63]. Butyrate can block NF-κB signaling via attenuating both the lipopolysaccharides-induced degradation/phosphorylation of IκBα and DNA binding of NF-κB and enhanced histone H3 acetylation [64]. The increase of the PEC-induced butyrate concentrations might be a potential mechanism for inhibiting the NF-κB pathway and NFkB-regulated inflammatory genes in the mucosa of pigs. This assumption may be supported by the present intra-associations between the expression of *NFKB* and related genes and relevance networking analysis with bacterial genera and microbial metabolites. Based on the present relevance networks, the PEC-related increase in moderate-to-high butyrate-producing bacterium may be involved in the suppression of pro-inflammatory gene and cytokine. Relevance network analysis shows that some SCFAs-producing bacteria, *Oscillibacter* and *Prevotella*, which were increased with the PEC diet, were important for the upregulation of MUC2 expression in the ileum (*Prevotella*), and downregulation of *TAK1* and *NFKB* expression in the cecum (*Oscillibacter*). *Oscillibacter* is associated with T-cell differentiation by enhancing and maintaining the IL-10-producing Treg cells [65]. However, increases in the *Prevotella* were accompanied by an increase of sIgA+ cells and relative expressions of *MUC2* in the intestinal mucosa of newborn piglets [66]. NF-κB pathway is a central regulatory pro-inflammatory signal transduction pathway. The present results of relevance network suggest that the anti-inflammatory effects of PEC may have been mediated by other pathogen-recognition receptors, apart from TLR-2 or TLR-4 [67]. Our results also suggest that enhanced SCFAs concentration could activate the nuclear transcription factors *AMPK*, which may serve as a negative regulator of NF-κB signaling [68,69]. In support of this possibility, C57BL/6 mice fed citrus PEC had an increased SCFAs production and an upregulated *AMPK* expression. This would be in line with present results [70]. Network analysis further indicated that butyrate-producing genera, including *Faecalibacterium*, *Roseburia* and *Blautia*, have strong associations with *AMPK* expression, a finding that has been described before [71,72]. However, relevance network analysis cannot explain whether the PEC-related beneficial effect was via a change in microbial structure and MAMP profile or via the alterations in SCFAs. Future studies are warranted to validate this finding and to confirm the true mechanism of the anti-inflammatory functionof PEC.

## 5. Conclusions

In summary, our results reveal that dietary intake of PEC had beneficial impacts on the intestinal health status of growing pigs. This was indicated by increased markers of mucus barrier function, reduced pro-inflammatory cytokine and increased abundances of potentially beneficial bacterial populations. Integrative analysis showed that the PEC-related alteration in the bacterial genera and fermentation metabolites, such as butyrate, were associated with the differentially expressed genes and cytokine in the ileum and cecum of pigs. These data offer a comprehensive insight into host–microbe interactions in the intestinal mucosa of swine and provide strong support for regulating the intestinal health of pigs and humans via dietary intervention with PEC and similar fermentable fibers.

## Figures and Tables

**Table 1:** Relative expression of genes related to luminal defense, barrier function, apoptosis and the innate immune signaling cascade in the ileum of pigs fed either the control (CON) or pectin (PEC) diet. (Mean values with their standard errors; *n* = 6/diet).

Genes of Interest	Control Diet	Pectin Diet	SEM	*p*
ZO-1	0.408	0.563	0.0961	0.281
Claudin-2	0.318	0.588	0.0828	0.044
Occludin	0.497	0.699	0.1034	0.196
MLCK	0.566	0.585	0.1227	0.912
MUC2	0.576	0.794	0.0256	0.087
MUC4	0.166	0.459	0.1397	0.170
TFF3	0.445	0.592	0.0761	0.205
bax	0.706	0.791	0.0638	0.366
BCL2	0.873	0.715	0.0677	0.122
caspase3	0.430	0.150	0.1102	0.159
TLR2	0.669	0.417	0.095	0.092
TLR4	0.783	0.592	0.157	0.140
NF-KB	0.524	0.224	0.0967	0.053
TRAF6	0.505	0.635	0.107	0.411
AMPK	0.567	0.592	0.092	0.852
TAK1	0.589	0.692	0.099	0.481
GPR41	0.690	0.606	0.118	0.627
GPR43	0.637	0.568	0.100	0.640

**Table 2:** Relative expression of genes related to luminal defense, barrier function, apoptosis and the innate immune signaling cascade in the cecum of pigs fed either the control (CON) or pectin (PEC) diet. (Mean values with their standard errors; n = 6/diet.).

Genes of Interest	Control Diet	Pectin Diet	SEM	*p*
ZO-1	0.619	0.609	0.0877	0.939
Claudin-2	0.478	0.688	0.0897	0.166
Occludin	0.644	0.517	0.1191	0.467
MLCK	0.595	0.455	0.0894	0.258
MUC2	0.257	0.558	0.0923	0.043
MUC4	0.409	0.507	0.1131	0.554
TFF3	0.401	0.770	0.0773	0.007
bax	0.541	0.426	0.0480	0.473
BCL2	0.539	0.770	0.1559	0.315
caspase3	0.155	0.467	0.1755	0.223
TLR2	0.559	0.459	0.0896	0.448
TLR4	0.539	0.706	0.0775	0.157
NF-KB	0.763	0.523	0.075	0.047
TRAF6	0.436	0.694	0.1018	0.290
AMPK	0.344	0.799	0.0735	0.001
TAK1	0.750	0.379	0.1114	0.040
GPR41	0.308	0.507	0.077	0.091
GPR43	0.330	0.430	0.093	0.466

## Supplementary Materials

Supplementary materials can be found at https://www.mdpi.com/2076-2607/8/4/535/s1. Figure S1: Butterfly plot showing the functional pathways predicted by PICURSt altered by diet in (A) ileum and (B) cecum of growing pigs provided PEC. Table S1: Composition of diets. Table S2: Oligonucleotide primer sequences used for qPCR. Table S3: Alpha-diversity of ileum and cecum gut microbiota from pigs fed PEC. Table S4: Alpha-diversity of ileum and cecum gut microbiota from pigs fed PEC. Table S5: Genera altered by diet in ileum and cecum of the growing pigs provided PEC.

## References

[1] Trompette A., Gollwitzer E.S., Yadava K., Sichelstiel A.K., Sprenger N., Ngom-Bru C., Blanchard C., Junt T., Nicod L.P., Harris N.L. (2014). Gut microbiota metabolism of dietary fiber influences allergic airway disease and hematopoiesis. Nat. Med..

[2] Bach Knudsen K.E., Lærke H.N., Hedemann M.S., Nielsen T.S., Ingerslev A.K., Gundelund Nielsen D.S., Theil P.K., Purup S., Hald S., Schioldan A.G. (2018). Impact of Diet-Modulated Butyrate Production on Intestinal Barrier Function and Inflammation. Nutrients.

[3] Frosali S., Pagliari D., Gambassi G., Landolfi R., Pandolfi F., Cianci R. (2015). How the Intricate Interaction among Toll-Like Receptors, Microbiota, and Intestinal Immunity Can Influence Gastrointestinal Pathology. J. Immunol. Res..

[4] Wang C., Li Q., Ren J. (2019). Microbiota-Immune Interaction in the Pathogenesis of Gut-Derived Infection. Front. Immunol..

[5] Kaiko G.E., Stappenbeck T.S. (2014). Host-microbe interactions shaping the gastrointestinal environment. Trends Immunol..

[6] Silva J.P.B., Navegantes-Lima K.C., Oliveira A.L.B., Rodrigues D.V.S., Gaspar S.L.F., Monteiro V.V.S., Moura D.P., Monteiro M.C. (2018). Protective Mechanisms of Butyrate on Inflammatory Bowel Disease. Curr. Pharm. Des..

[7] Ang Z., Ding J.L. (2016). GPR41 and GPR43 in Obesity and Inflammation - Protective or Causative?. Front. Immunol..

[8] Zou T., Wei W., Cao S., Zhang H., Liu J.J.A. (2020). Effects of Dietary Fat Sources during Late Gestation on Colostrum Quality and Mammary Gland Inflammation in Lipopolysaccharide-Challenged Sows. Animals.

[9] Chen C.-H., Sheu M.-T., Chen T.-F., Wang Y.-C., Hou W.-C., Liu D.-Z., Chung T.-C., Liang Y.-C. (2006). Suppression of endotoxin-induced proinflammatory responses by citrus pectin through blocking LPS signaling pathways. Biochem. Pharmacol..

[10] Jiang T., Gao X., Wu C., Tian F., Lei Q., Bi J., Xie B., Wang H.Y., Chen S., Wang X. (2016). Apple-derived pectin modulates gut microbiota, improves gut barrier function, and attenuates metabolic endotoxemia in rats with diet-induced obesity. Nutrients.

[11] Hino S., Sonoyama K., Bito H., Kawagishi H., Aoe S., Morita T. (2013). Low-methoxyl pectin stimulates small intestinal mucin secretion irrespective of goblet cell proliferation and is characterized by jejunum Muc2 upregulation in rats. J. Nutr..

[12] Johansson M.E., Phillipson M., Petersson J., Velcich A., Holm L., Hansson G.C. (2008). The inner of the two Muc2 mucin-dependent mucus layers in colon is devoid of bacteria. Proc. Natl. Acad. Sci. USA.

[13] Van der Sluis M., De Koning B.A., De Bruijn A.C., Velcich A., Meijerink J.P., Van Goudoever J.B., Büller H.A., Dekker J., Van Seuningen I., Renes I.B. (2006). Muc2-deficient mice spontaneously develop colitis, indicating that MUC2 is critical for colonic protection. Gastroenterology.

[14] Koh A., De Vadder F., Kovatcheva-Datchary P., Bäckhed F. (2016). From Dietary Fiber to Host Physiology: Short-Chain Fatty Acids as Key Bacterial Metabolites. Cell.

[15] Tian L., Bruggeman G., van den Berg M., Borewicz K., Scheurink A.J.W., Bruininx E., de Vos P., Smidt H., Schols H.A., Gruppen H. (2017). Effects of pectin on fermentation characteristics, carbohydrate utilization, and microbial community composition in the gastrointestinal tract of weaning pigs. Molecular Nut. Food Res..

[16] Chung W.S.F., Meijerink M., Zeuner B., Holck J., Louis P., Meyer A.S., Wells J.M., Flint H.J., Duncan S.H. (2017). Prebiotic potential of pectin and pectic oligosaccharides to promote anti-inflammatory commensal bacteria in the human colon. FEMS Microbiol. Ecol..

[17] Onumpai C., Kolida S., Bonnin E., Rastall R.A. (2011). Microbial utilization and selectivity of pectin fractions with various structures. Appl. Environ. Microbiol..

[18] Munukka E., Rintala A., Toivonen R., Nylund M., Yang B., Takanen A., Hanninen A., Vuopio J., Huovinen P., Jalkanen S. (2017). *Faecalibacterium prausnitzii* treatment improves hepatic health and reduces adipose tissue inflammation in high-fat fed mice. ISME J..

[19] Fang W., Zhang L., Meng Q., Wu W., Lee Y.K., Xie J., Zhang H. (2018). Effects of dietary pectin on the profile and transport of intestinal bile acids in young pigs. J. Anim. Sci..

[20] Zhang L., Wu W., Lee Y.K., Xie J., Zhang H.J.F.I.M. (2018). Spatial Heterogeneity and Co-occurrence of Mucosal and Luminal Microbiome across Swine Intestinal Tract. Front. Microbiol..

[21] Council N.R. (2012). Nutrient Requirements of Swine.

[22] Wu W., Xie J., Zhang H. (2016). Dietary fibers influence the intestinal SCFAs and plasma metabolites profiling in growing pigs. Food Funct..

[23] Livak K.J., Schmittgen T.D. (2001). Analysis of relative gene expression data using real-time quantitative PCR and the 2(-Delta Delta C(T)) Method. Methods.

[24] Metzler-Zebeli B.U., Lawlor P.G., Magowan E., Zebeli Q. (2018). Interactions between metabolically active bacteria and host gene expression at the cecal mucosa in pigs of diverging feed efficiency. J. Anim. Sci..

[25] Wu W., Zhang L., Xia B., Tang S., Liu L., Xie J., Zhang H. (2020). Bioregional Alterations in Gut Microbiome Contribute to the Plasma Metabolomic Changes in Pigs Fed with Inulin. Microorganisms.

[26] Caporaso J.G., Kuczynski J., Stombaugh J., Bittinger K., Bushman F.D., Costello E.K., Fierer N., Peña A.G., Goodrich J.K., Gordon J.I. (2010). QIIME allows analysis of high-throughput community sequencing data. Nat. Methods.

[27] Langille M.G.I., Zaneveld J., Caporaso J.G., McDonald D., Knights D., Reyes J.A., Clemente J.C., Burkepile D.E., Vega Thurber R.L., Knight R. (2013). Predictive functional profiling of microbial communities using 16S rRNA marker gene sequences. Nat Biotechnol.

[28] Love M.I., Huber W., Anders S. (2014). Moderated estimation of fold change and dispersion for RNA-seq data with DESeq2. Genome Biol..

[29] Mcmurdie P.J., Holmes S. (2014). Waste Not, Want Not: Why Rarefying Microbiome Data Is Inadmissible. PLoS Comput. Biol..

[30] Benjamini Y., Hochberg Y. (1995). Controlling the False Discovery Rate: A Practical and Powerful Approach to Multiple Testing. J. R. Stat. Soc. Ser. B Methodol..

[31] Lê Cao K.-A., González I., Déjean S. (2009). tegrOmics: An R package to unravel relationships between two omics datasets. Bioinformatics.

[32] LêCao K.-A., Martin P.G.P., Robert-Granié C., Besse P. (2009). Sparse canonical methods for biological data integration: Application to a cross-platform study. BMC Bioinform..

[33] Zhang L., Fan X., Zhong Z., Xu G., Shen J. (2015). Association of plasma diamine oxidase and intestinal fatty acid-binding protein with severity of disease in patient with heat stroke. Am. J. Emerg. Medlicine.

[34] Wang J., Zeng L., Tan B., Li G., Huang B., Xiong X., Li F., Kong X., Liu G., Yin Y. (2016). Developmental changes in intercellular junctions and Kv channels in the intestine of piglets during the suckling and post-weaning periods. J. Anim. Sci. Biotechnol..

[35] Liu B., Zhang J., Sun P., Yi R., Han X., Zhao X. (2019). Raw Bowl Tea(Tuocha) Polyphenol Prevention of Nonalcoholic Fatty Liver Disease by Regulating Intestinal Function in Mice. Biomolecules.

[36] Wiese M. (2019). The potential of pectin to impact pig nutrition and health: Feeding the animal and its microbiome. FEMS Microbiol. Lett..

[37] Xu R., Lu Y., Wang J., Liu J., Su Y., Zhu W. (2019). Effects of the different dietary fibers on luminal microbiota composition and mucosal gene expression in pig colons. J. Funct. Foods.

[38] Albenberg L.G., Wu G.D. (2014). Diet and the intestinal microbiome: Associations, functions, and implications for health and disease. Gastroenterology.

[39] Marteyn B., Scorza F.B., Sansonetti P.J., Tang C. (2011). Breathing life into pathogens: The influence of oxygen on bacterial virulence and host responses in the gastrointestinal tract. Cell. Microbiol..

[40] Hughes E.R., Winter M.G., Duerkop B.A., Spiga L., Furtado de Carvalho T., Zhu W., Gillis C.C., Büttner L., Smoot M.P., Behrendt C.L. (2017). Microbial Respiration and Formate Oxidation as Metabolic Signatures of Inflammation-Associated Dysbiosis. Cell Host Microbe.

[41] Shin N.-R., Whon T.W., Bae J.-W. (2015). Proteobacteria: Microbial signature of dysbiosis in gut microbiota. Trends Biotechnol..

[42] Jena P.K., Sheng L., Liu H.-X., Kalanetra K.M., Mirsoian A., Murphy W.J., French S.W., Krishnan V.V., Mills D.A., Wan Y.-J.Y. (2017). Western Diet-Induced Dysbiosis in Farnesoid X Receptor Knockout Mice Causes Persistent Hepatic Inflammation after Antibiotic Treatment. Am. J. Pathol..

[43] Sanders M.E., Merenstein D.J., Reid G., Gibson G.R., Rastall R.A. (2019). Probiotics and prebiotics in intestinal health and disease: From biology to the clinic. Nat. Rev. Gastroenterol. Hepatol..

[44] Wu G.D., Chen J., Hoffmann C., Bittinger K., Chen Y.-Y., Keilbaugh S.A., Bewtra M., Knights D., Walters W.A., Knight R.J.S. (2011). Linking long-term dietary patterns with gut microbial enterotypes. Science.

[45] Shahi S.K., Freedman S.N., Gibson-Corley K.N., Karandikar N.J., Murray J., Mangalam A.K. (2019). *Prevotella histicola*, a human gut commensal, is as potent as Copaxone® in an animal model of multiple sclerosis. Front. Immunol..

[46] Leknoi Y., Mongkolsuk S., Sirikanchana K. (2017). Assessment of swine-specific bacteriophages of *Bacteroides fragilis* in swine farms with different antibiotic practices. J. Water Health.

[47] Arnold M., Crienen A., Swam H., von Berg S., Jolie R., Nathues H. (2019). Prevalence of *Lawsonia intracellularis* in pig herds in different European countries. Porc. Health Manag..

[48] Wang Y., Wang Y., Sun L., Grenier D., Yi L. (2018). *Streptococcus suis* biofilm: Regulation, drug-resistance mechanisms, and disinfection strategies. Appl. Microbiol. Biotechnol..

[49] Hwang S., Gwon S.-Y., Kim M.S., Lee S., Rhee K.-J. (2013). *Bacteroides fragilis* toxin induces IL-8 secretion in HT29/C1 cells through disruption of E-cadherin junctions. Immune Netw..

[50] Castaño-Rodríguez N., Kaakoush N.O., Lee W.S., Mitchell H.M. (2017). Dual role of *Helicobacter* and *Campylobacter* species in IBD: A systematic review and meta-analysis. Gut.

[51] Rivera-Chávez F., Zhang L.F., Faber F., Lopez C.A., Byndloss M.X., Olsan E.E., Xu G., Velazquez E.M., Lebrilla C.B., Winter S. (2016). Depletion of butyrate-producing *Clostridia* from the gut microbiota drives an aerobic luminal expansion of Salmonella. Cell Host Microbe.

[52] McKenzie C., Tan J., Macia L., Mackay C.R.J.I.R. (2017). The nutrition-gut microbiome-physiology axis and allergic diseases. Immunol. Rev..

[53] Neurath M.F. (2017). Current and emerging therapeutic targets for IBD. Nat. Rev. Gastroenterol. Hepatol..

[54] Yeh J.-Y., Ga A.R. (2018). Systemic cytokine response in pigs infected orally with a *Lawsonia intracellularis* isolate of South Korean origin. J. Vet. Med. Sci..

[55] Nogueira M.G., Collins A.M., Donahoo M., Emery D. (2013). Immunological responses to vaccination following experimental *Lawsonia intracellularis* virulent challenge in pigs. Vet. Microbiol..

[56] Sun Y., He Y., Wang F., Zhang H., de Vos P., Sun J. (2017). Low-methoxyl lemon pectin attenuates inflammatory responses and improves intestinal barrier integrity in caerulein-induced experimental acute pancreatitis. Mol. Nutr. Food Res..

[57] Kim Y.S., Ho S.B. (2010). Intestinal goblet cells and mucins in health and disease: Recent insights and progress. Curr. Gastroenterol. Rep..

[58] Ottman N., Davids M., Suarez-Diez M., Boeren S., Schaap P.J., dos Santos V.A.M., Smidt H., Belzer C., de Vos W.M. (2017). Genome-scale model and omics analysis of metabolic capacities of *Akkermansia muciniphila* reveal a preferential mucin-degrading lifestyle. Appl. Environ. Microbiol..

[59] Cooke C.L., An H.J., Kim J., Canfield D.R., Torres J., Lebrilla C.B., Solnick J.V. (2009). Modification of gastric mucin oligosaccharide expression in rhesus macaques after infection with Helicobacter pylori. Gastroenterology.

[60] Myhill L.J., Stolzenbach S., Hansen T.V., Skovgaard K., Stensvold C.R., Andersen L.O.B., Nejsum P., Mejer H., Thamsborg S.M., Williams A.R. (2018). Mucosal barrier and Th2 immune responses are enhanced by dietary inulin in pigs infected with Trichuris suis. Front. Immunol..

[61] Zhang S., Hu H., He W., Muhammad Z., Pan S. (2019). Regulatory Roles of Pectin Oligosaccharides on Immunoglobulin Production in Healthy Mice Mediated by Gut Microbiota. Mol. Nutr. Food Res..

[62] Wu W., Sun M., Chen F., Cao A.T., Liu H., Zhao Y., Huang X., Xiao Y., Yao S., Zhao Q. (2017). Microbiota metabolite short-chain fatty acid acetate promotes intestinal IgA response to microbiota which is mediated by GPR43. Mucosal Immunol..

[63] Chu H., Mazmanian S.K. (2013). Innate immune recognition of the microbiota promotes host-microbial symbiosis. Nat. Immunol..

[64] Lee C., Kim B.G., Kim J.H., Chun J., Im J.P., Kim J.S. (2017). Sodium butyrate inhibits the NF-kappa B signaling pathway and histone deacetylation, and attenuates experimental colitis in an IL-10 independent manner. Int. Immunopharmacol..

[65] Arpaia N., Campbell C., Fan X., Dikiy S., van der Veeken J., Deroos P., Liu H., Cross J.R., Pfeffer K., Coffer P.J. (2013). Metabolites produced by commensal bacteria promote peripheral regulatory T-cell generation. Nature.

[66] Hu L., Geng S., Li Y., Cheng S., Fu X., Yue X., Han X. (2018). Exogenous Fecal Microbiota Transplantation from Local Adult Pigs to Crossbred Newborn Piglets. Front. Microbiol..

[67] Zhang Q., Lenardo M.J., Baltimore D. (2017). 30 Years of NF-κB: A Blossoming of Relevance to Human Pathobiology. Cell.

[68] Pongkorpsakol P., Satitsri S., Wongkrasant P., Chittavanich P., Kittayaruksakul S., Srimanote P., Chatsudthipong V., Muanprasat C. (2017). Flufenamic acid protects against intestinal fluid secretion and barrier leakage in a mouse model of Vibrio cholerae infection through NF-κB inhibition and AMPK activation. Eur. J. Pharmacol..

[69] Elamin E.E., Masclee A.A., Dekker J., Pieters H.-J., Jonkers D.M. (2013). Short-chain fatty acids activate AMP-activated protein kinase and ameliorate ethanol-induced intestinal barrier dysfunction in Caco-2 cell monolayers. J. Nutr..

[70] Shtriker M.G., Hahn M., Taieb E., Nyska A., Moallem U., Tirosh O., Madar Z. (2018). Fenugreek galactomannan and citrus pectin improve several parameters associated with glucose metabolism and modulate gut microbiota in mice. Nutrition.

[71] Si X., Shang W., Zhou Z., Strappe P., Wang B., Bird A., Blanchard C. (2018). Gut Microbiome-Induced Shift of Acetate to Butyrate Positively Manages Dysbiosis in High Fat Diet. Mol. Nut. Food Res..

[72] Henning S.M., Yang J., Hsu M., Lee R.-P., Grojean E.M., Ly A., Tseng C.-H., Heber D., Li Z. (2018). Decaffeinated green and black tea polyphenols decrease weight gain and alter microbiome populations and function in diet-induced obese mice. Eur. J. Nutr..

