# Supplementary material for: Modulation of Pectin on Mucosal Innate Immune Function in Pigs Mediated by Gut Microbiota
Source: Microorganisms. 2020 Apr 8;8(4):535. doi: 10.3390/microorganisms8040535 (PMC7232157; doi:10.3390/microorganisms8040535)
Supplement: Supplementary file 1 [file microorganisms-08-00535-s001.zip › Supplemental Table 1.docx]

Table S1: Composition of diets^1^.

| Items | CON | PEC |
| --- | --- | --- |
| Ingredients (% as-fed) | | |
| Corn | 57.92 | 52.92 |
| Soybean meal (44% CP) | 28.87 | 28.87 |
| Soybean oil | 4.81 | 4.81 |
| L-lysine HCl | 0.05 | 0.05 |
| Monocalcium phosphate (21%) | 1.37 | 1.37 |
| Limestone | 0.68 | 0.68 |
| Salt | 0.30 | 0.30 |
| Premix | 1.00 | 1.00 |
| pectin | - | 5.00 |
| Analyzed composition (% DM) | | |
| Digestible energy (kcal kg^-1^) | 3300 | 3300 |
| Crude Protein | 18.52 | 18.52 |
| Acid-hydrolyzed fat | 7.30 | 7.30 |
| Ca | 0.65 | 0.65 |
| P | 0.56 | 0.56 |

1. All diets were formulated at Institute of Animal science, Chinese Academy of Agricultural Sciences.
2. Vitamin and mineral premixes were supplied per kg of diet as-fed: Mn (MnSO_4_·H_2_O), 12.57 mg; Zn (ZnSO_4_), 314.2 mg; Cu (CuSO_4_·5H2O), 24.0 mg; Se (Na_2_SeO_3_), 40.0 mg; Fe (FeSO_4_), 100.0 mg; I (KI), 0.5 mg; choline, 600.0 mg; vitamin A (all-trans retinyl acetate), 2.84 mg; vitamin D3, 0.22 mg; a-tocopherol, 40 mg; phylloquinone, 2.0 mg; thiamin, 1.0 mg; riboflavin, 5.0 mg; nicotinic acid, 15.0 mg; pantothenic acid, 15.0 mg; pyridoxine, 2.0 mg; vitamin B12, 0.025 mg; folic acid, 2 mg; biotin, 0.2 mg.
