# Supplementary material for: Modulation of Pectin on Mucosal Innate Immune Function in Pigs Mediated by Gut Microbiota
Source: Microorganisms. 2020 Apr 8;8(4):535. doi: 10.3390/microorganisms8040535 (PMC7232157; doi:10.3390/microorganisms8040535)
Supplement: Supplementary file 1 [file microorganisms-08-00535-s001.zip › Supplemental Table 2.docx]

| Gene | Accession number | Primer sequences (5'-3') | Size,bp |
| --- | --- | --- | --- |
| ZO-1 | XM_005659811.1 | F: CTCCAGGCCCTTACCTTTCG | 248 |
|  |  | R: GGGGTAGGGGTCCTTCCTAT |  |
| Claudin-2 | NM_001161638.1 | F: GCATCATTTCCTCCCTGTT | 156 |
|  |  | R: TCTTGGCTTTGGGTGGTT |  |
| Occlaudin | NM_001163647.2 | R: GGGGTAGGGGTCCTTCCTAT |  |
|  |  | F: CAGGTGCACCCTCCAGATTG |  |
| MLCK | XM_021070241.1 | F:TGACCCTGTCCTGGTATGG | 111 |
|  |  | R:GGCATGTGGCTAGTTCCTT |  |
| MUC2 | [XM_021082584.1](https://www.ncbi.nlm.nih.gov/entrez/viewer.fcgi?db=nucleotide&id=1191854901) | F: CGCATGGATGGCTGTTTCTG | 145 |
|  |  | R: ATTGCTCGCAGTTGTTGGTG |  |
| MUC4 | XM_001926442.1 | F: GATGCCCTGGCCACAGAA | 89 |
|  |  | R: TGATTCAAGGTAGCATTCATTTGC |  |
| TFF3 | [NM_001243483.1](https://www.ncbi.nlm.nih.gov/entrez/viewer.fcgi?db=nucleotide&id=343478294) | F:GGGAAAAAGCTCCCTGGCTA | 86 |
|  |  | R:TCAAGGGTCACGGAAAGTGG |  |
| bax | [XM_013998624.2](https://www.ncbi.nlm.nih.gov/entrez/viewer.fcgi?db=nucleotide&id=1191886809) | F:AAGCGCATTGGAGATGAACT | 121 |
|  |  | R:TGCCGTCAGCAAACATTTC |  |
| BCL2 | [XM_021099593.1](https://www.ncbi.nlm.nih.gov/entrez/viewer.fcgi?db=nucleotide&id=1191808555) | F:TGCCTTTGTGGAGCTGTATG | 144 |
|  |  | R:GCCCGTGGACTTCACTTATG |  |
| caspase3 | XM 005671704.2 | F:GGAATGGCATGTCGATCTGGT | 351 |
|  |  | R:ACTGTCCGTCTCAATCCCAC |  |
| TLR2 | NM_213761.1 | F:TAAAGACGGTGTGCTGCAAG | 76 |
|  |  | R:CAATCCCCAAGACCCATGCT |  |
| TLR4 | [NM_001293316.1](https://www.ncbi.nlm.nih.gov/entrez/viewer.fcgi?db=nucleotide&id=648216105) | F:AAGGTTATTGTCGTGGTGT | 179 |
|  |  | R:CTGCTGAGAAGGCGATAC |  |
| NF-KB | NM_001048232.1 | F: GGCTACCCTGGCACAGAAAT | 72 |
|  |  | R: TTGGAAGGAGCAGCACTCAG |  |
| TRAF6 | XM_005652801.2 | F: TGGGTAGTCTTCGGAGGGAG | 72 |
|  |  | R: AAACAAGGCGGGGATGCTAT |  |
| AMPK | NM_214266.1 | F:GCTCCTTGACCGGAAGCA | 86 |
|  |  | R:CAGACTGGCACACATTTCGAA |  |
| TAK1 | NM_001114280·1 | F:TGCCCAAACTCCAAAGAATC | 151 |
|  |  | R:TTTGCTGGTCCTTTTCATCC |  |
| GPR41 | NM_001315601.1 | F:GCCCTTGCCCTTCATCTTCT | 136 |
|  |  | R:CCGGGTCTTGTACCAGAGTG |  |
| GPR43 | NM_001278758.1 | F: CTGCCTGGGATCGTCTGTG | 249 |
|  |  | R: CATACCCTCGGCCTTCTGG |  |
| β-actin | [XM_003357928.4](https://www.ncbi.nlm.nih.gov/entrez/viewer.fcgi?db=nucleotide&id=1191819600) | F: GCGTAGCATTTGCTGCATGA | 252 |
|  |  | R: GCGTGTGTGTAACTAGGGGT |  |
| GAPDH | [NM_001206359.1](https://www.ncbi.nlm.nih.gov/entrez/viewer.fcgi?db=nucleotide&id=329744641) | F: CGTGTCGGTTGTGGATCTGA | 209 |
|  |  | R: TGACGAAGTGGTCGTTGAGG |  |

Supplemental Table 2. Oligonucleotide primer sequences used for qPCR
