# Supplementary material for: Modulation of Pectin on Mucosal Innate Immune Function in Pigs Mediated by Gut Microbiota
Source: Microorganisms. 2020 Apr 8;8(4):535. doi: 10.3390/microorganisms8040535 (PMC7232157; doi:10.3390/microorganisms8040535)
Supplement: Supplementary file 1 [file microorganisms-08-00535-s001.zip › Supplemental Table 3.docx]

Table S3: Alpha-diversity of ileum and cecum gut microbiota from pigs fed pectin

| Taxanomic Level | Diversity Index | Diet | Location | Interaction |
| --- | --- | --- | --- | --- |
| Phylum | Chao1 | 0.17 | 0.32 | 0.08 |
|  | Shannon | 0.18 | 0.81 | 0.48 |
| Class | Chao1 | 0.08 | <0.01 | 0.06 |
|  | Shannon | 0.09 | 0.64 | 0.61 |
| Order | Chao1 | 0.13 | <0.01 | 0.03 |
|  | Shannon | 0.02 | 0.06 | 0.43 |
| Family | Chao1 | 0.11 | <0.01 | 0.03 |
|  | Shannon | 0.33 | 0.11 | 0.32 |
| Genus | Chao1 | <0.01 | 0.01 | 0.03 |
|  | Shannon | 0.09 | 0.07 | 0.25 |
