# Supplementary material for: Modulation of Pectin on Mucosal Innate Immune Function in Pigs Mediated by Gut Microbiota
Source: Microorganisms. 2020 Apr 8;8(4):535. doi: 10.3390/microorganisms8040535 (PMC7232157; doi:10.3390/microorganisms8040535)
Supplement: Supplementary file 1 [file microorganisms-08-00535-s001.zip › Supplemental Table 4.docx]

Table S4: Alpha-diversity of ileum and cecum gut microbiota from pigs fed pectin

| Taxonomic Level | Location | Diversity Index | Con | Pec | *P* |
| --- | --- | --- | --- | --- | --- |
| Phylum | Ileum | Chao1 | 17.0 | 10.5 | 0.17 |
| Phylum | Cecum | Chao1 | 12.0 | 11.2 | 0.36 |
| Phylum | Ileum | Shannon | 1.17 | 0.80 | 0.12 |
| Phylum | Cecum | Shannon | 1.08 | 1.00 | 0.43 |
| Class | Ileum | Chao1 | 27.5 | 21.0 | 0.08 |
| Class | Cecum | Chao1 | 19.2 | 18.2 | 0.68 |
| Class | Ileum | Shannon | 1.77 | 1.33 | 0.07 |
| Class | Cecum | Shannon | 1.60 | 1.40 | 0.18 |
| Order | Ileum | Chao1 | 50.0 | 28.7 | 0.13 |
| Order | Cecum | Chao1 | 35.4 | 26.5 | 0.48 |
| Order | Ileum | Shannon | 2.19 | 1.45 | 0.06 |
| Order | Cecum | Shannon | 1.65 | 1.43 | 0.21 |
| Family | Ileum | Chao1 | 85.3 | 62.3 | 0.11 |
| Family | Cecum | Chao1 | 49.6 | 48.2 | 0.58 |
| Family | Ileum | Shannon | 2.68 | 1.75 | 0.04 |
| Family | Cecum | Shannon | 1.99 | 1.72 | 0.31 |
| Genus | Ileum | Chao1 | 181.7 | 145.9 | 0.02 |
| Genus | Cecum | Chao1 | 138.6 | 133.9 | 0.39 |
| Genus | Ileum | Shannon | 3.06 | 1.99 | 0.13 |
| Genus | Cecum | Shannon | 3.21 | 2.96 | 0.51 |

Values are median. *P*-values obtain from Kruskal-Wallis Test. Medians without a common letter differ.
