# Supplementary material for: Modulation of Pectin on Mucosal Innate Immune Function in Pigs Mediated by Gut Microbiota
Source: Microorganisms. 2020 Apr 8;8(4):535. doi: 10.3390/microorganisms8040535 (PMC7232157; doi:10.3390/microorganisms8040535)
Supplement: Supplementary file 1 [file microorganisms-08-00535-s001.zip › Supplemental Table 5.docx]

Table S5a**:** Ileal mucosa genera altered by diet in growing pigs provided pectin.^1^

| *Taxonomy^2^* | | Inu Relative to Con | |
| --- | --- | --- | --- |
| Phylum | Genus | Fold Difference^3^ | *P*^4^ |
| *Actinobacteria* |  |  |  |
|  | *Rothia* | -5.53 | ≤0.01 |
|  | *Propionibacterium* | -6.60 | ≤0.01 |
|  | *Corynebacterium_1* | -5.02 | ≤0.01 |
|  | *Kocuria* | -3.28 | ≤0.05 |
|  | *Lysinimonas* | -5.02 | ≤0.05 |
|  | *Collinsella* | -2.69 | ≤0.05 |
| *Bacteroidetes* |  |  |  |
|  | *Bacteroides* | -3.82 | ≤0.01 |
|  | *Parabacteroides* | -5.20 | ≤0.01 |
|  | *Barnesiella* | -5.89 | ≤0.01 |
|  | *Empedobacter* | 1.61 | ≤0.05 |
|  | *Chryseobacterium* | 1.62 | ≤0.05 |
|  | *Porphyromonas* | 2.45 | ≤0.05 |
|  | *Dyadobacter* | 2.34 | ≤0.05 |
| *Firmicutes* |  |  |  |
|  | *Staphylococcus* | -4.33 | ≤0.01 |
|  | *Bacillus* | 1.84 | ≤0.01 |
|  | *Lachnospiraceae_NK4A136_group* | -2.13 | ≤0.01 |
|  | *Enterococcus* | -4.24 | ≤0.01 |
|  | *Anaerostipes* | -4.14 | ≤0.01 |
|  | *Lactococcus* | 1.79 | ≤0.01 |
|  | *Oceanobacillus* | 1.92 | ≤0.01 |
|  | *Megamonas* | -3.00 | ≤0.05 |
|  | *Brochothrix* | 1.99 | ≤0.05 |
|  | *Ruminiclostridium_5* | 5.76 | ≤0.05 |
|  | *Exiguobacterium* | -4.45 | ≤0.05 |
|  | *Ruminococcaceae_UCG_013* | -2.97 | ≤0.05 |
| *Fusobacteria* |  |  |  |
|  | *Cetobacterium* | -6.50 | ≤0.01 |
| *Gemmatimonadetes* |  |  |  |
|  | *Gemmatimonas* | 2.64 | ≤0.01 |
| *Proteobacteria* |  |  |  |
|  | *Brevundimonas* | 2.19 | ≤0.01 |
|  | *Aquitalea* | -7.16 | ≤0.01 |
|  | *Aeromonas* | -6.12 | ≤0.01 |
|  | *Campylobacter* | -2.32 | ≤0.01 |
|  | *Sphingopyxis* | 2.45 | ≤0.01 |
|  | *Aquabacterium* | 2.16 | ≤0.01 |
|  | *Ralstonia* | 2.43 | ≤0.01 |
|  | *Hyphomicrobium* | -5.42 | ≤0.01 |
|  | *Stenotrophomonas* | 2.14 | ≤0.01 |
|  | *Moraxella* | 1.74 | ≤0.01 |
|  | *Woodsholea* | -6.17 | ≤0.01 |
|  | *Novosphingobium* | 1.94 | ≤0.01 |
|  | *Ochrobactrum* | -4.76 | ≤0.01 |
|  | *Dechloromonas* | 2.08 | ≤0.05 |
|  | *Blastomonas* | 2.12 | ≤0.05 |
|  | *Pseudoalteromonas* | -5.50 | ≤0.05 |
|  | *Lawsonia* | -5.19 | ≤0.05 |
|  | *Pelomonas* | 1.91 | ≤0.05 |
|  | *Pseudoxanthomonas* | -4.86 | ≤0.05 |
|  | *Helicobacter* | -3.19 | ≤0.05 |
|  | *Psychrobacter* | 1.56 | ≤0.05 |
|  | *Parasutterella* | -4.09 | ≤0.05 |
|  | *Rhodobacter* | -4.56 | ≤0.05 |
|  | *Rahnella* | 1.77 | ≤0.05 |
|  | *Bosea* | 1.66 | ≤0.05 |
| *Tenericutes* |  |  |  |
|  | *Mycoplasma* | -3.96 | ≤0.01 |
| *Verrucomicrobia* |  |  |  |
|  | *Akkermansia* | -4.92 | ≤0.01 |

^1^ Pairwise comparisons of diet group using Negative Binomial Wald Test.

^2^ Taxonomy assignments based on Silva Database.

^3^ Log2 fold differences.

^4^ Benjamini and Hochberg FDR correction.

Table S5b**:** Cecal mucosa genera altered by diet in growing pigs provided pectin.^1^

| *Taxonomy^2^* | | Inu Relative to Con | |
| --- | --- | --- | --- |
| Phylum | Genus | Fold Difference^3^ | *P*^4^ |
| *Bacteroidetes* |  |  |  |
|  | *Bacteroides* | -4.78 | ≤0.01 |
|  | *Alloprevotella* | 0.49 | ≤0.05 |
|  | *Prevotella* | 2.28 | ≤0.05 |
| *Chlamydiae* |  |  |  |
|  | *Chlamydia* |  |  |
| *Firmicutes* |  |  |  |
|  | *Candidatus_Soleaferrea* | -2.85 | ≤0.01 |
|  | *Subdoligranulum* | -1.94 | ≤0.01 |
|  | *Staphylococcus* | -1.76 | ≤0.01 |
|  | *Anaerofilum* | -1.64 | ≤0.01 |
|  | *Streptococcus* | -1.44 | ≤0.05 |
|  | *Catenibacterium* | -1.40 | ≤0.05 |
|  | *Allisonella* | -1.31 | ≤0.05 |
|  | *Anaerovibrio* | -0.76 | ≤0.05 |
|  | *Blautia* | 0.74 | ≤0.05 |
|  | *Anaerotruncus* | 0.85 | ≤0.01 |
|  | *Coprococcus_1* | 1.21 | ≤0.01 |
|  | *Lachnoclostridium* | 1.45 | ≤0.01 |
|  | *Oscillibacter* | 1.64 | ≤0.01 |
|  | *Ruminiclostridium* | 2.17 | ≤0.05 |
|  | *Faecalibacterium* | 3.90 | ≤0.05 |
|  | *Clostridium_sensu_stricto_6* | 4.66 | ≤0.05 |
|  | *Defluviitaleaceae_UCG_011* | -3.08 | ≤0.05 |
|  | *Eubacterium_ruminantium_group* | -2.12 | ≤0.05 |
|  | *Eubacterium_coprostanoligenes_group* | -1.48 | ≤0.05 |
|  | *Lachnospiraceae_UCG_004* | 2.10 | ≤0.05 |
| *Proteobacteria* |  |  |  |
|  | *Lawsonia* | -8.29 | ≤0.01 |
|  | *Aeromonas* | -4.39 | ≤0.01 |
|  | *Actinobacillus* | -3.52 | ≤0.05 |
|  | *Escherichia_Shigella* | -2.10 | ≤0.05 |
|  | *Campylobacter* | 0.81 | ≤0.05 |
|  | *Desulfurivibrio* | 0.86 | ≤0.01 |
|  | *Arcobacter* | 0.90 | ≤0.05 |
|  | *Thalassospira* | 2.90 | ≤0.01 |
|  | *Anaeroplasma* | 2.75 | ≤0.01 |

^1^ Pairwise comparisons of diet group using Negative Binomial Wald Test.

^2^ Taxonomy assignments based on Silva Database.

^3^ Log2 fold differences.

^4^ Benjamini and Hochberg FDR correction.
